# G-tube contrast check: Transition from fluoroscopy to abdominal radiographs

**DOI:** 10.21203/rs.3.rs-5632134/v1

**Published:** 2024-12-24

**Authors:** Muhammad Hameed, Charles James, Kevin Wong, Paul Lewis, Paula Roberson, Kelli Schmitz, Sateesh Jayappa, Amy Rowell, Marcene McVay-Gilam, Todd Frost, Adam Springer, Mary Moore

**Affiliations:** University of Arkansas for Medical Sciences; University of Arkansas for Medical Sciences; University of Arkansas for Medical Sciences; University of Arkansas for Medical Sciences; University of Arkansas for Medical Sciences; University of Arkansas for Medical Sciences; University of Arkansas for Medical Sciences; Arkansas Children’s Hospital; University of Arkansas for Medical Sciences; Arkansas Children’s Hospital; KLS Physics Group, LLC; University of Arkansas for Medical Sciences

## Abstract

**Background:**

Reports of radiographic exam evaluation for G-tube malposition in children are limited.

**Objective:**

Evaluate effectiveness of a new 2-view abdominal radiograph exam protocol instituted to provide 24/7 coverage at 2 affiliated hospitals and replace the prior fluoroscopic G-tube contrast check exam.

**Materials and Methods:**

G-tube radiographic exams performed between December 2019 and May 2022 at 2 affiliated hospitals were identified and retrospective chart review was performed to delineate exam test yield, accuracy, sensitivity, specificity. Additional data collected included exam adherence to protocol, years of experience of the reporting pediatric radiologist, reporting time, and 30-day adverse events.

**Results:**

227 exams were performed in 186 patients. 2-view radiograph protocol was followed in 81.9% (186/227); Additional radiograph views were performed in 18.1% (41/227); additional contrast volume in 9.3% (21/227). Reporting time under 1 hour occurred in 79.7% (181/227). 5.7% (13/227) exams were reported as indeterminate adding a median time delay of 40 minutes (IQR 90). Indeterminate exam reporting did not correlate with years of experience of the reporting pediatric radiologist (p=0.19); reporting time over 1 hour occurred more often in the after-hours group (p= 0.032). Fluoroscopic G-tube contrast check was requested in 8 of 13 indeterminate readings. Following reclassification of indeterminate exams based on clinical suspicion, test performance: yield 94.3%, accuracy 97.3%, sensitivity 81.8%, specificity 98.2%, PPV 69.2%, NPV 99.1%,

**Conclusion:**

This new diagnostic exam performed well with high test yield, accuracy, specificity and negative predictive value. The exam mostly followed protocol, allowed timely resumption of G-tube use, and provided a needed 24/7 remote coverage option for the new affiliated hospital.

## Introduction

During the current workforce shortage of pediatric radiologists and radiologic technologists across North America, remote reads of diagnostic imaging exams has been increasing [1,2]. This trend comes at a time with increasing hospital leadership expectation for evening/overnight final reads of diagnostic imaging exams [3]. Imaging check of a replaced gastrostomy tube (G-tube) has traditionally been a fluoroscopic exam performed by a pediatric radiologist. Introduction of a diagnostic abdominal radiograph exam following protocolled contrast administration as an option to replace selected fluoroscopic exams has been reported at a single institution [4]. Though an unpublished SCORCH survey in 2019 showed many centers tending from a fluoroscopic exam to a radiographic exam (personal communication), validation of G-tube radiographic exam performance at other institutions is lacking. We report our experience replacing the fluoroscopic G-tube check exam with a 2-view abdominal radiograph exam with protocolled contrast administration for two affiliated children’s hospitals separated by 3 hours driving distance. Need for such a radiographic protocol arose at our institution when we were unable to provide 24/7 fluoroscopic coverage of potentially malpositioned G-tubes at the remote children’s hospital which was staffed initially with one pediatric radiologist; after-hours exams in these patients previously encountered diagnostic delay awaiting pediatric radiologist availability for an on-site fluoroscopic exam or required a 3-hour ambulance transport of the patient to the established children’s hospital with 24/7 pediatric radiologist coverage for a fluoroscopic G-tube check exam.

## Methods

Institutional review board approval was obtained to create a REDCap database and retrospectively study of all G-tube abdominal radiograph exams performed with a new protocol established to replace onsite fluoroscopic G-tube check by a pediatric radiologist. The radiographic exams were performed between December 2019 and May 2022 at an established children’s hospital (Institution A) and new affiliated community children’s hospital (Institution B), separated by 3 hours driving distance. Radiographic examinations were identified in a shared picture archiving and communications system [PACS] (Fuji Synapse PACS) with by the procedure description: XR G-TUBE CHECK W CONTAST ABD 2VW.

### Radiographic exam protocol:

**Figure 1:**
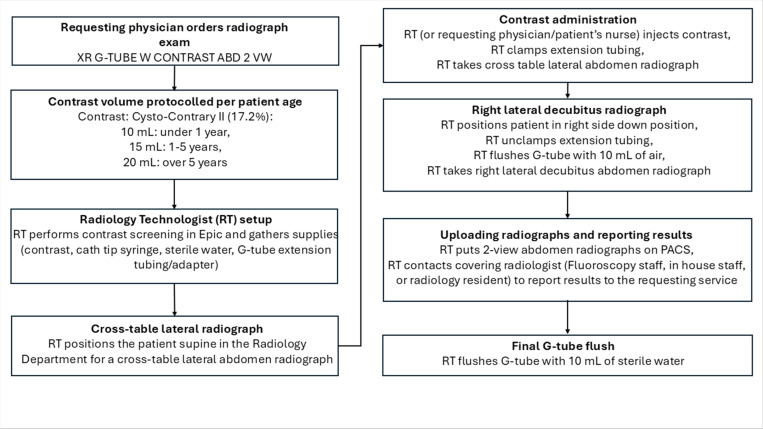
G-tube radiographic exam protocol.

**Figure 2:**
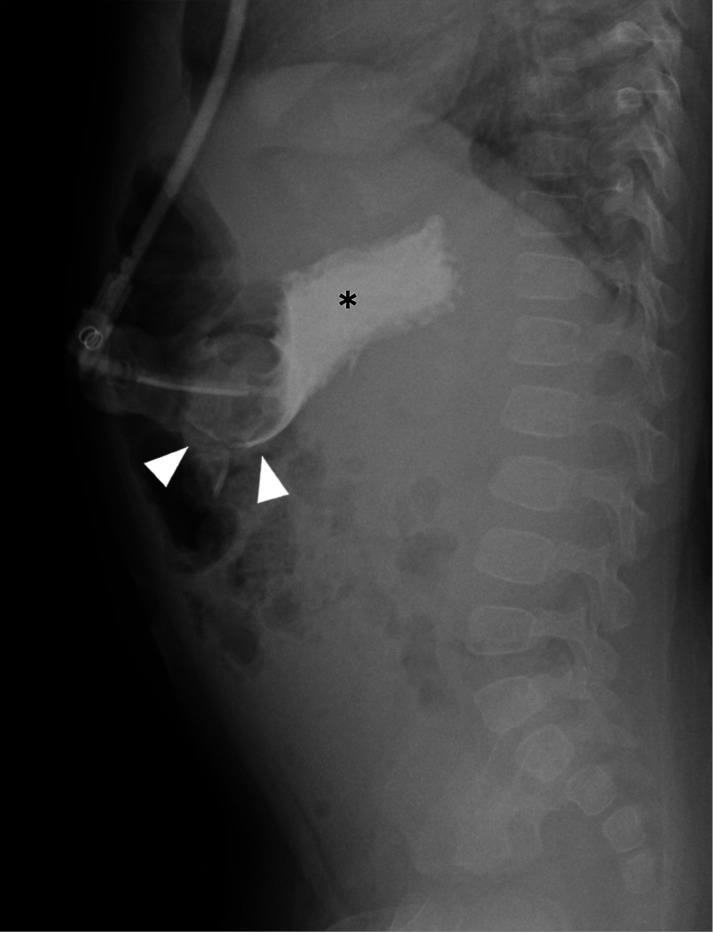
Cross-table lateral abdomen radiograph shows intraluminal contrast flow into the gastric lumen (*) and gastric contrast outlining the tube retention balloon (arrowheads).

**Figure 3:**
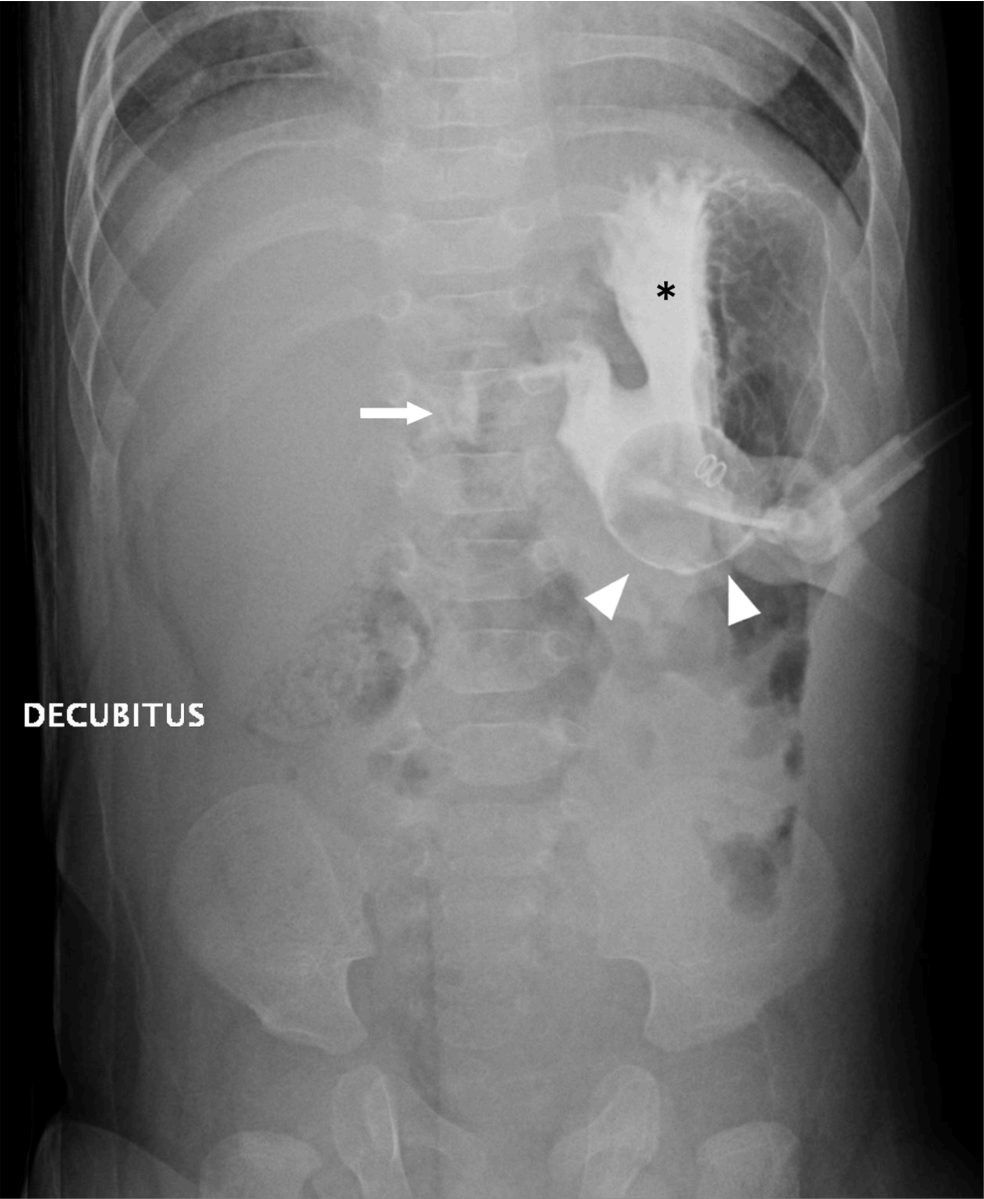
Frontal radiograph with the patient in a right lateral decubitus position shows gastric luminal contrast (*) and proximal duodenal contrast (arrow). The gastric luminal contrast outlines the G-tube retention balloon (arrowheads).

The radiographic exam protocol (Fig. 1) included a two-view abdominal radiograph exam following contrast administration (Cysto-Conray II, 17.2%) by the radiology technologist (RT) protocolled based on patient’s age: 10 mL under 1-year, 15 mL for 1 to 5 years, and 20 mL over 5 years (the RT defers to the requesting physician or patient’s nurse if resistance to contrast injection is encountered). A cross-table lateral radiograph (Fig. 2) is followed by a frontal radiograph with the patient in a right lateral decubitus position (Fig. 3). Upon exam completion, the RT contacted the pediatric radiologist covering the Fluoroscopy work assignment during normal weekday hours (Monday-Friday 8:00am-5:00 pm) or the on-call pediatric radiologist covering Fluoroscopy via a phone call, beeper page or a Secure Chat message in Epic. The reviewing pediatric radiologist could request additional views and/or additional contrast on an ad-hoc basis. If the pediatric radiologist was not available for a timely interpretation (after-hours) the covering overnight radiology resident entered a preliminary report note in Epic which was subsequently reviewed by the reporting pediatric radiologist. Each subject’s radiographic exam was dictated using a reporting template (Nuance PowerScibe 360) commenting on contrast flow, G-tube retention component alignment, peritoneal air and peritoneal contrast. During a temporary shortage of Cysto-Conray II, 17.2% during the study period, Cystografin-Dilute, 18% served as a backup contrast in some exams.

The report result was subsequently classified in the REDCap database as positive (abnormal exam with abnormal G-tube retention component alignment, suspicious peritoneal air, or extraluminal contrast leakage), negative (normal exam with normal intraluminal contrast flow and normal G-tube retention component alignment) or indeterminate (equivocal G-tube retention component alignment or uncertainty of intraluminal contrast) based on the report wording in Epic. To determine exam sensitivity and specificity, indeterminate exams were reclassified based on clinical suspicion, a method suggested by several researchers [5,6]. Specifically, when an indeterminate exam was accompanied by low clinical suspicion of G-tube malposition/malfunction by the consulted Pediatric Surgery team or requesting service, no further imaging was performed and the study reclassified as a negative (normal) radiographic exam. When there was high clinical suspicion of G-tube malfunction (such as pain with G-tube use) by the consulted Pediatric Surgery team or requesting service, a fluoroscopic G-tube contrast check was requested and the radiographic study reclassified as a positive (abnormal) radiographic exam.

### Fluoroscopic exam protocol:

For indeterminate radiographic exams with high clinical suspicion, a referring physician ordered a Fluoro G-tube contrast check and the RT contacted the pediatric radiologist to perform an on-site exam. The pediatric radiologist traveled to the fluoroscopy suite from home or an on-site assignment and protocolled the exam contrast in Epic (Cysto-Conray II, 17.2%, Barium sulfate, 40%, or Cystografin-Dilute, 18%). This pediatric radiologist then obtained fluoroscopic images following injection of contrast through the G-tube (administrated by the pediatric radiologist or RT) in both lateral and frontal positions as directed by the pediatric radiologist. The exam was tailored to confirm intraluminal contrast flow (gastric or gastroduodenal per pediatric radiologist judgement), confirm appropriate G-tube retention component alignment within the gastric lumen, and detect any extraluminal contrast leakage. The G-tube was then flushed with 10 mL of sterile water and the images sent to PACS.

Data collection from chart review was entered into a REDCap database and included demographics (age, weight, biologic sex), clinical indication, and referral location (Table 1). Additional data collected included number of radiograph views, contrast volume administered, and exam reporting times (under 20 minutes, 20 to 60 minutes, 1 to 2 hours, and over 2 hours). Reporting time was measured from the moment the images are uploaded into PACS to the time when the pediatric radiologist completed their exam report; for after-hours exams with a radiology resident preliminary report, entry of this note in Epic was used to calculate reporting time. Reporting time was further categorized as under or over 1 hour. Reporting times over 1 hour was compared between exams performed during daytime hours (Monday-Friday, 8:00 am-5:00 pm) and those performed after-hours.

Any additional time delay for indeterminate exams was calculated as the time between the reporting of the radiograph exam as indeterminate to the onset of the fluoroscopic exam or resumption of G-tube use based on low clinical suspicion. After-hours exams performed at the newer affiliated community hospital that were read remotely by pediatric radiologists at the established children’s hospital were identified as lack of this radiographic exam may have required an ambulance transport to the established children’s hospital 3 hours away. Indeterminate exam readings by pediatric radiologist with less experience (5 years or less) were compared to indeterminate exam readings by pediatric radiologists with greater experience (over 5 years).

To confirm a negative radiographic exam, chart review was performed for the 30-day period following the radiographic exam looking for any return ED visits/hospitalizations, imaging studies, Surgery team notes (progress notes, consults, operative notes) or Interventional Radiology (IR) procedures to assess for a potentially missed G-tube abnormality or related 30-day adverse event. The positive (abnormal) G-tube radiographic exams were acted on accordingly by a subspecialist (Pediatric Surgery team, Pediatric IR team) to obtain a normal G-tube alignment and resume G-tube use. Chart reviews were performed by MYH, CAJ, KW, PSL. All indeterminate exams and exams with protocol variance were further reviewed at additional chart review by a pediatric radiologist with 30 years of experience (CAJ) to discern the potential reason for an indeterminate report and to suggest potential reasons for the additional radiograph views or additional contrast administration. Data availability statement: Retrospective study data can be made available upon reasonable request.

### Effective radiation dose comparison:

Comparative radiation dose information was collected on the 8 study patients undergoing both the G-tube radiographic exam and a fluoroscopic G-tube contrast check exam. Data collected from PACS included number of radiograph exam views, kVp, patient thickness for each radiographic view, dose area product (DAP). This data with the patient weight was provided to a medical physicist to calculate an effective dose for both the radiographic and fluoroscopic exams. Specifically, the energy imparted ε (Equation 1) was calculated for each radiographic view and each fluoroscopic procedure [7]. The coefficient ω(z) in Equation 1 depends on on half-value layer (HVL) of the Xray beam as well as model-based coefficients α and β that account for patient thickness z and X-ray tube potential (kVp) (Equation 2) [8]. The coefficients α and β were interpolated for each radiographic view based on the kVp and the AP or lateral dimensions as measured on images on PACS. For fluoroscopic exams, the average of each patient’s lateral and frontal thickness was used with the average kVp of the case to interpolate α and β. The energy imparted was then converted to effective dose) by multiplying by region-specific conversion factors and scaling for patient mass ω(M) (Equation 3) [8]. The conversion factor for left lateral stomach (13.1 mSv/J) was used for cross-table lateral views while the factor for AP stomach (40.2 mSv/J) was used for frontal views (decubitus or supine). The effective doses from radiographic views were summed to determine the effective dose for each subject.

Equation 1 ε=ω(z)×DAPJ

Equation 2 $$\omega \left(z\right)=\alpha \times HVL+\beta \text{J}{\text{R}}^{-1}{\text{cm}}^{-2}$$

Equation 3 $$E=\varepsilon \times (\frac{E}{\varepsilon }{)}_{i}\times \frac{70.9}{M}\text{mSv}$$

### Statistical analyses:

Diagnostic test characteristics [yield, accuracy, sensitivity, specificity, positive predictive value (PPV), and negative predictive value (NPV)] were calculated according to standard formulae [9] excluding the 13 exams reported as indeterminate, and again after reclassifying the indeterminate exams based on clinical suspicion, as described in Methods and Discussion. The association between indeterminate reports and staff experience (five years or less versus over five years) as well as the association of after-hours exams with reporting times (one hour or less versus over one hour) were assessed by chi-square using the software package StatXact-12 (version 12.0; Cytel Studio, Cytel Inc., 2019). P ≤ 0.05 was considered statistically significant.

## Results

227 exams were performed in 186 patients over the 2.5 years study period. Patient demographic details, referral location, and clinical indication are provided in Table 1. The standard 2-view radiograph protocol was followed in 81.9% (186/227) of the cases (Table 2), with 2 of these exams, 1.1% (2/186) reported as positive (abnormal). The remaining 18.1% (41/227) of radiographic exams required additional views which yielded 3 additional positive (abnormal exams); including additional views, 2.2% (5/227) of radiographic exams were positive (abnormal) at initial analysis. At retrospective review of additional radiograph views (Table 2), a majority appeared to be performed for diagnostic purposes (confirm G-tube retention component alignment or duodenal contrast flow) and less commonly for technical factors (radiographic image coverage, faint administered contrast, radiograph exposure issue) or a protocol deviation (RT incorrectly performed a scout view). In the views obtained for additional diagnostic information 93.5% (29/31) were performed to confirm intraluminal G-tube retention component alignment (n=15) or to document duodenal contrast flow (n=14). 78.9% (179/227) of the exams adhered to the contrast volume protocol. Contrast volume over the protocolled volume was administered in 9.3% (21/227) of the studies (Table 2), including RT error (n=12), lack of contrast outlining G-tube retention component (n=6), and faint contrast on the 2-view radiographs (n=3). 11.5% (26/227) lacked documentation in the medical record regarding administered contrast volume.

Findings on true positive exams included a G-tube retention component in the abdominal wall or superficial gastrostomy tract anterior to the stomach (n=3), intraperitoneal contrast leakage (n=1), large pneumoperitoneum (n=1), and a small amount of extraluminal air (n=1). Free extraluminal (peritoneal) air was seen on 4 exams, all within 7 days of primary G-tube placement. In one of these subjects the free air was large volume in a symptomatic patient and surgical revision was required. In a second subject the small amount of extraperitoneal free air was noted with extraluminal contrast leakage in a clinically declining patient and surgical revision was required. In the 2 other subjects, the post-operative free air was small and of no clinical significance.

The overall test yield was 94.3% (214/227), with a normal or abnormal exam reported (Table 3). 13 exams were reported as indeterminate, all relating to uncertainty of G-tube retention component alignment within the gastric lumen. The indeterminate exams had a median time delay of 40 minutes (IQR 90). Indeterminate exam reporting did not correlate with years of experience as a pediatric radiologist (p=0.19). Of the 13 indeterminate exams, 4 had no follow-up imaging (low clinical suspicion) and were reclassified as true negatives. Eight indeterminate exams underwent an additional fluoroscopic G-tube check. Four of these exams revealed malpositioned G-tubes and were reclassified as true positives. Four indeterminate exams with high clinical suspicion underwent fluoroscopy, which confirmed normal G-tube placement and were reclassified as false positives. In the remaining indeterminate exam, the Surgery team was consulted for G-tube site leakage/irritation in a patient in the intensive care unit with acute respiratory failure, sepsis and renal failure. The surgery consult (finalized 19 minutes before the radiographic exam was reported) noted the radiographic exam showed the tube appears in place with contrast in bowel loops, but added the final Radiology reading was pending. The radiographic exam (reported over 2 hours after the exam was uploaded into PACS) noted indeterminate G-tube retention balloon alignment and small bowel contrast, without gastric luminal contrast. Considerations in the report impression included fast contrast transit into the small bowel or transpyloric G-tube retention component malposition. The Surgery team did not order a fluoroscopic G-tube contrast check in this particular patient who one day later had high nasogastric tube output with bright red blood. The GI team was consulted 2 days after the radiographic exam and endoscopy showed the gastric retention balloon was in the duodenal bulb causing gastric outlet obstruction; this indeterminate radiographic exam was classified as a false negative as the clinical suspicion of the Surgery team did not lead to a fluoroscopic G-tube check. One other radiographic exam initially read as normal had G-tube retention component tract malposition identified in retrospect at our monthly pediatric radiology group Peer-Review conference and was classified as a false negative (Fig. 9); this autistic patient returned to the Emergency Department 7 days after the radiographic exam with increased abdominal pain and leakage at the ostomy site and G-tube retention component malposition was confirmed on repeat G-tube abdominal radiograph exam.

Excluding indeterminate results, the exam performance metrics were as follows: sensitivity 83.3%, specificity 100%, positive predictive value (PPV) 100%, negative predictive value (NPV) 99.5%, and accuracy 99.5%. After reclassifying the indeterminate exams based on clinical suspicion, the performance metrics were: sensitivity 81.8%, specificity 98.2%, PPV 69.2%, and NPV 99.1% (Table 3). Test accuracy (true positives + true negatives/total exams) was 99.5% when excluding indeterminate results and decreased slightly to 97.3% after reclassification of indeterminate exams.

Exam reporting time included 40.5% (92/227) under 20 minutes, 39.2% (89/227) within 20 to 60 minutes, % (30/227) within 1 to 2 hours, and 6.6% (15/227) over 2 hours. Reporting time occurred under 1 hour in 79.7% (181/227). Reporting time over 1 hour occurred more often in the after-hours group (p= 0.032). Radiology resident preliminary readings were found in 36.1% (82/227) of the exams, concurring with the final pediatric radiologist report in 96.3% (79/82) of exams. 5.7% (13/227) exams were reported as indeterminate adding a median time delay of 40 minutes (IQR 90). 10 exams performed after-hours at Institution B (the newer remote community hospital with limited after-hours on-site Fluoro coverage) were interpreted remotely at the established children’s hospital, Institution A, thereby potentially avoiding a 3-hour inter-hospital ground transport.

Concerning radiation exposure, comparative effective doses in the 8 patients with both radiographic exams and fluoroscopic exams shows the typical 2-view radiographic exam can result in approximately half the effective dose of a fluoroscopic G-tube check in some patients (Table 4). However, when additional radiographs were requested by the reporting pediatric radiologist, the radiographic exam effective dose was 1.5 to 6.9 times higher than the fluoroscopic exam.

## Discussion

This new radiographic exam was efflciently incorporated by radiology technologists and pediatric radiologists across two departments separated by 3 hours diving distance. 24/7 exam reporting was provided in a timely fashion at two hospitals during a time of pediatric radiologist and radiology technologist workforce shortage. This new diagnostic exam performed well with high test yield, accuracy, specificity and negative predictive value.

The current shortage of pediatric radiologists across North American is expected to continue into future years as the number of pediatric radiology fellowship trainees has been decreasing for several years and an advancing age of pediatric radiologists nearing retirement [10, 11]. This pediatric radiologist shortage is compounded by a shortage of radiologic technologists available to facilitate diagnostic radiology exams [2]. This shortage of radiology subspecialists comes at a time when leadership of children’s hospitals are having a greater expectation of 24/7 final reads of imaging exams as well expectations of coverage for a greater number of affiliated institutions [12, 13]. During the Covid-19 pandemic, remote reporting of pediatric radiology exams experienced a marked increase, as evidenced by a Society of Chiefs of Radiology at Children’s Hospitals (SCORCH) survey, where 95.8% of groups now incorporate remote reading work assignments into their staffing schedules, up from 50% prior to the pandemic [1].

At our institution a change from the established fluoroscopic contrast check of a G-tube by an on-site pediatric radiologist was necessitated after a new affiliated community children’s hospital opened separated by 3 hours driving distance from the established affiliated children’s hospital. With only 1 pediatric radiologist on staff at the new community hospital at onset of this project, 24/7 coverage for an on-site fluoroscopic exam was not feasible; delay awaiting pediatric radiologist availability for an on-site fluoroscopic exam or a 3-hour ambulance transport to the established children’s hospital for a fluoroscopic exam would have been required. Prior to the protocol, such “after-hours” exams which would have usually required a pediatric radiologist traveling into the hospital for an on-site fluoroscopic G-tube check. This study validates and the prior single institution report of radiograph exam performance where patients received either a radiograph exam or a fluoroscopic exam for both G-tube and GJ-tube (gastrojejunostomy) checks based on availability of an attending radiologist in the hospital [4]. At our institution, the radiograph exam was the initial imaging exam for all G-tube checks; potential GJ-tube malfunctions at our institution are assessed by the interventional radiology (IR) service and are not included in this radiograph exam protocol.

**Figure 4:**
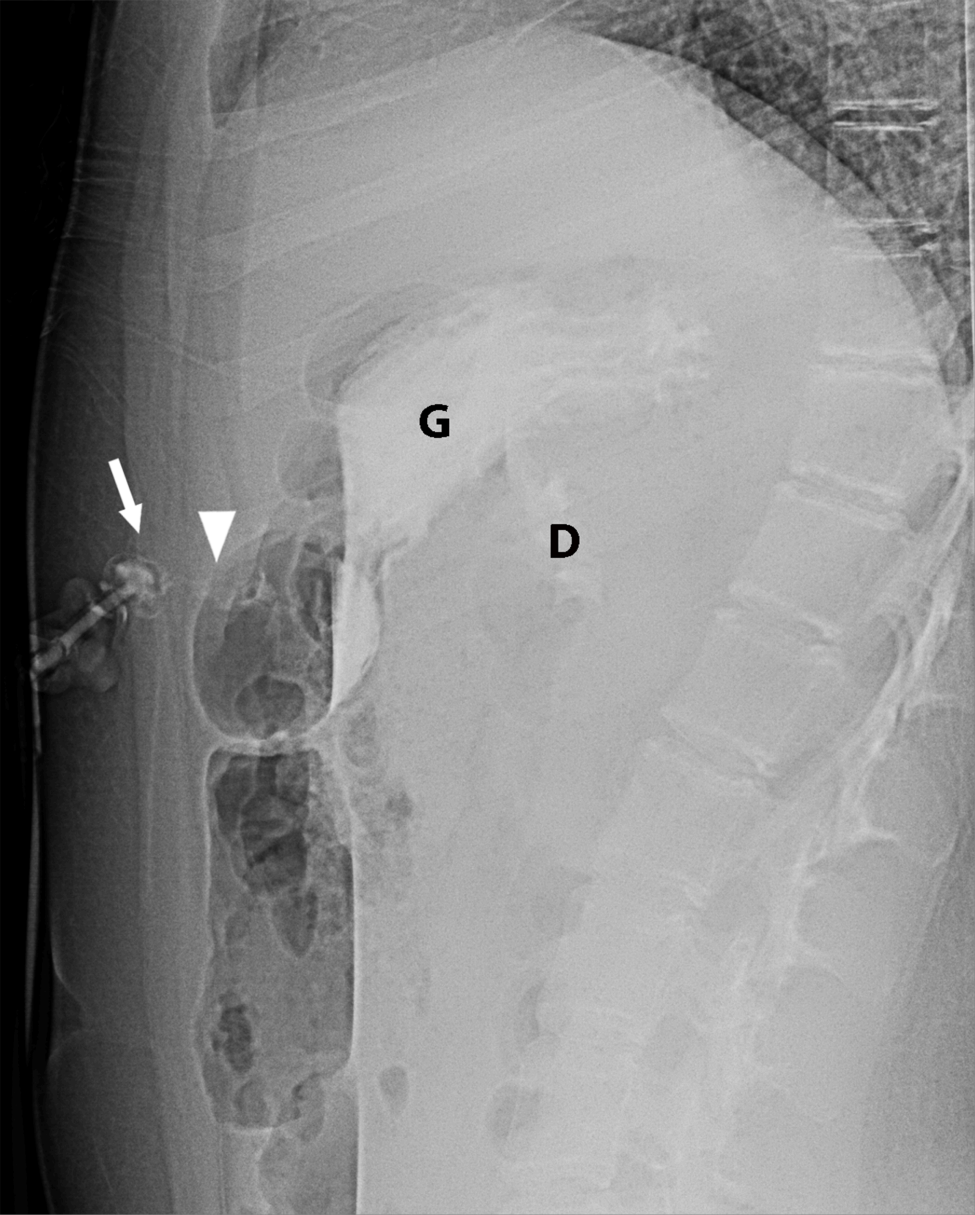
Cross-table abdominal radiograph shows gastric luminal contrast flow (G) and contrast in the proximal duodenum (D). However, the G-tube retention component (arrow) projects in the anterior abdominal wall in the superficial gastrostomy tract (arrowhead).

The pediatric radiologists sought input from Emergency Department and the Pediatric Surgery service on protocol development given their involvement in a majority of patients needing a G-tube check or replacement. Regarding the 2-view abdominal radiograph exam, the cross table lateral view was chosen to look for pneumoperitoneum, G-tube alignment relative to the gastric lumen (Fig. 3), and intraluminal contrast flow. The subsequent frontal radiograph with the patient in a right side down position was chosen to optimize detecting duodenal contrast flow (Fig. 4). An age-based protocol for volume of contrast to administer was devised to simplify this step for the radiology technologist (RT) performing the study, as an updated weight might be lacking in the medical record when the study is ordered.

**Figure 5:**
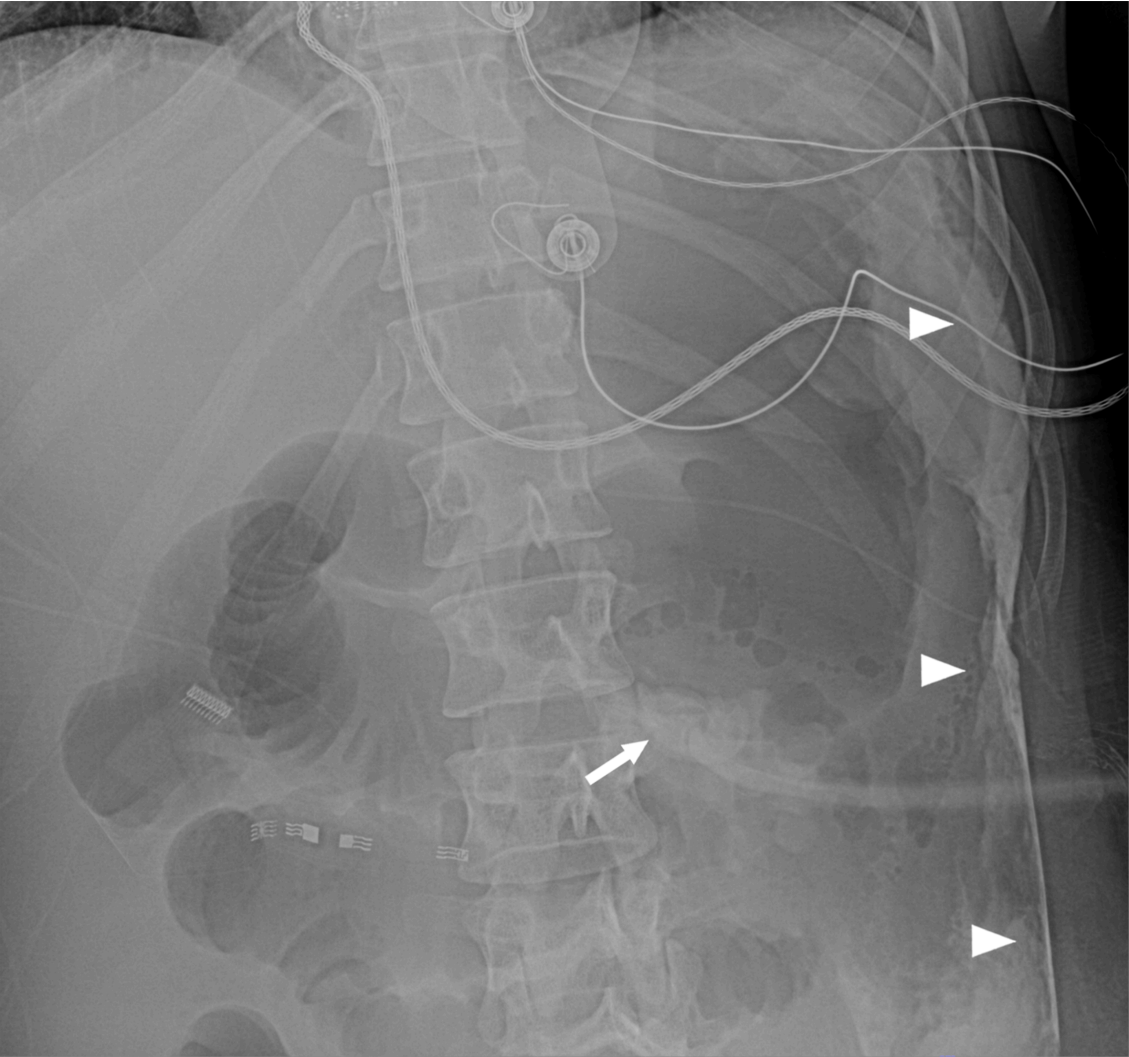
Frontal radiograph with the patient in a right lateral decubitus position shows the G-tube (arrow) overlying the greater curvature of the stomach. Peritoneal contrast leakage (arrowheads) is noted in the left lateral abdomen.

Indeterminate exams mainly related to uncertainty of position of the G-tube retention component in the gastric lumen or the gastrostomy tract (Fig. 5). Publications reporting the performance of a diagnostic study often exclude or mishandle the indeterminate exams. In fact, one report by Shinkins et al. found that only 35% of published studies reviewed reported indeterminate results accurately [5]. Excluding indeterminate exams falsely elevates exam accuracy and performance parameters as seen in our Results. Handling of indeterminate results is a complex issue and our search found no commonly agreed upon method for handling these data. The most pertinent reference we could find lists clinical suspicion as a reasonable approach to reclassify indeterminate exams [5]. In addition, Item 15 in the Standard for Reporting Diagnostic Accuracy (STARD) 2015 guidelines as reported by Cohen, et al also supports this option [6]. Specifically, if an indeterminate exam had high clinical suspicion (such as pain during G-tube use), a fluoroscopic G-tube contrast check exam was performed and the abdominal radiograph exam reclassified as a positive (abnormal) exam. If the indeterminate abdominal radiograph exam had low clinical suspicion, no fluoroscopic exam was performed and the radiograph exam was reclassified as a negative (normal) exam. Note that the Surgery team or other requesting service may feel an exam reported by a pediatric radiologist as indeterminate is normal on their independent review of the exam images or the patient tolerated resumption of G-tube use without any concerning symptoms. After this secondary analysis including all exams, the new abdominal radiograph exam performed well with particularly high accuracy (97.3%), specificity (98.2%), and negative predictive value (99.1%). This study builds on the prior report of G-tube radiographic exam performance reported (75% sensitivity and 100% specificity) in a larger number of patients (227 versus 126), while effectively managing indeterminate exams [4].

Deviations from the prescribed 2-view exam protocol occurred with RT error (incorrect view, scout view incorrectly obtained), technical factors (inadequate radiograph coverage/exposure issue) or when the interpreting radiologists asked for additional radiographs or additional contrast to improve the diagnostic information of the exam. Given equivocal gastric intraluminal alignment of the G-tube retention component on all 13 indeterminate exams, consideration of a different view [left lateral decubitus view (LLD)] may be a helpful consideration; this LLD view was performed in only 5 patients in this study (Table 2). On-site after-hours fluoroscopic studies decreased with introduction of this new exam, which was well received by our pediatric radiologists providing overnight fluoroscopy call coverage. Though both protocols outlined above have similar RT set-up steps, the fluoroscopic G-tube check exam requires the RT to contact the pediatric radiologist covering the fluoroscopy assignment; this pediatric radiologist travels to the fluoroscopy room from an on-site work assignment or from home after-hours. Of note 36.1% (82/227) of exams had an initial radiology resident preliminary reading note in Epic; without the radiographic exam option, a significant proportion of these would have required a pediatric radiologist on overnight call to drive to the hospital for an on-site fluoroscopic exam. The exam transition from a fluoroscopic exam to a more streamlined radiographic exam was well received by our radiologic technologists who were also short staffed during the study period. No 30-day adverse events resulted from this diagnostic exam transition.

Exam reporting time was studied given the relation to resumption of G-tube use for nutrition, hydration and medications in a timely fashion. While less than half of the exams met the initial protocol reporting time goal of 20 minutes, 79.7% (181/227) of exams were reported under 1 hour, allowing timely resumption of G-tube use for hydration, medications and nutrition in a majority of patients. Delayed reporting of exams (over 1 hour) occurred more often in the after-hours group, when pediatric radiologist coverage is reduced. Delay in reporting time can impact clinical management as seen in a false negative exam in this study, where the Surgery team felt the G-tube alignment was acceptable on their review of the radiographs before the radiology report was finalized; the Radiology report subsequently suggested possible transpyloric alignment of the G-tube which caused gastric outlet obstruction in this patient.

### Limitations

Limitations of this study start with the inherent limitations of retrospective chart review. For instance, when reviewing the reason for additional radiograph views, a given patient may have had overlapping need for more diagnostic information in addition to technical limitation of the initial 2-view exam (faint administered contrast). In regards to protocol adherence, the amount of actual contrast administered by the RT was not documented in an Epic note in 26 of the 227 exams. A limitation affecting exam reporting time occurred as the study period was before our pediatric radiology group converted to 24/7 attending pediatric radiologist reads of diagnostic radiology exams; a portion of study exams included an overnight preliminary reading of radiology residents. Another change at our institution after the study period, has been elimination of beeper paging system; pediatric radiologists are now messaged via the Epic Secure Chat for timely reporting of selected radiographic exams which may have an impact on reporting time. A future study assessing timelier exam reporting is warranted given our group’s transition to 24/7 coverage and change in contacting a pediatric radiologist after this 2.5-year study period.

Limitations exist with calculating effective radiation doses in our small number of patients (n = 8) that had both exams. One factor is that the DAP includes radiation from the entire beam, while the patient may not intercept the entire beam. Adjusting the DAP to account for any air gap would decrease the effective dose estimates. Also, the patient thickness measured on radiographs may be magnified, contributing to artificially high effective dose estimates. Lastly, the FDA does not regulate the accuracy of DAP meters for radiographic nor fluoroscopic units and they are not routinely tested for accuracy.

This new exam was efflciently incorporated by radiology technologists and pediatric radiologists in departments on two campuses, as well as by radiology residents. The same standard of care was provided and reported at two hospitals separated by 3 hours diving distance during a time of pediatric radiologist and radiology technologist workforce shortage.

## Conclusion

This new diagnostic exam performed well with high test yield, accuracy, specificity and negative predictive value. The exam mostly followed protocol, allowed timely resumption of G-tube use, and provided the needed remote coverage option for a new affiliated hospital.

## Supplementary Material

Supplement 1

## Figures and Tables

**Figure 6:**
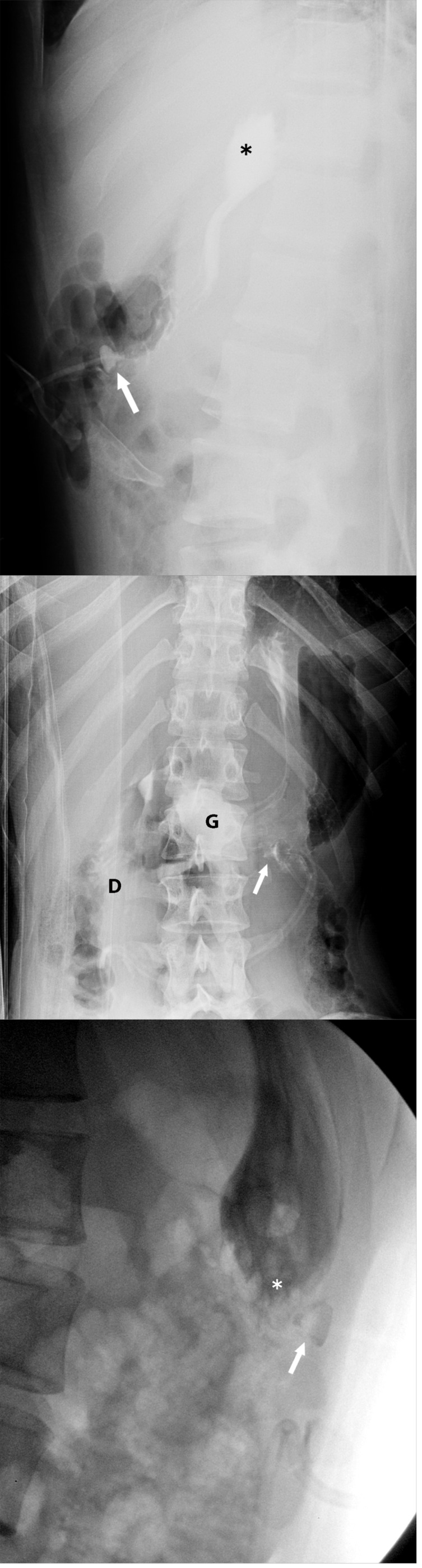
17-year-old female with indeterminate abdomen radiograph exam and high clinical suspicion who underwent a fluoroscopic G-tube contrast check exam confirming a true positive (abnormal exam). **a** Cross-table lateral abdomen radiograph shows intraluminal contrast flow into the gastric fundus (*). Alignment of the G-tube retention component (arrow) within the gastric lumen is indeterminate. **b** Right lateral decubitus abdomen radiograph shows definitive intraluminal contrast flow into the gastric lumen (G) and duodenum (D). Alignment of the G-tube retention component (arrow) within the gastric lumen is uncertain. **c** Left posterior oblique view during fluoroscopic G-tube contrast check shows the G-tube retention component (arrow) outside of the gastric luminal contrast (*). **d** Right posterior oblique fluoroscopic view (tangential view to Fig. 8) confirms extraluminal alignment of the G-tube retention component (arrow) relative to the gastric luminal contrast (*) administered under fluoroscopy.

**Figure 7:**
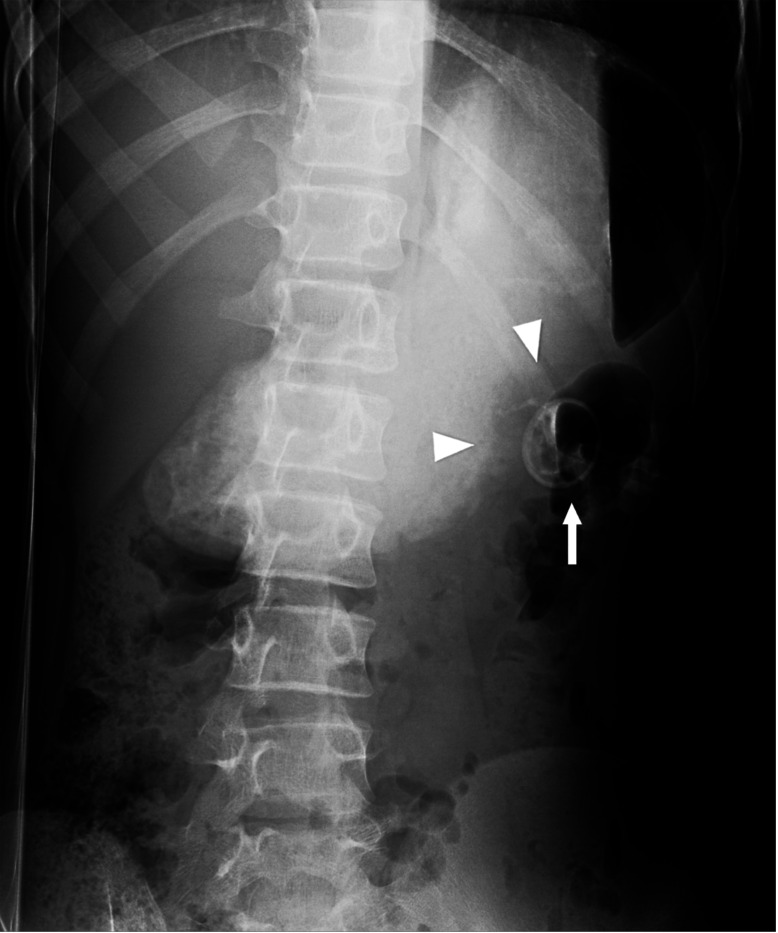
Frontal radiograph with the patient in a right lateral decubitus position shows separation between the G-tube retention component (arrow) and gastric luminal contrast (arrowheads). This was exam was retrospectively classified as a false negative exam at pediatric radiology group peer-review.
