# Supplementary material for: G-tube contrast check: Transition from fluoroscopy to abdominal radiographs
Source: Res Sq. 2024 Dec 24:rs.3.rs-5632134. Preprint. [Version 1] doi: 10.21203/rs.3.rs-5632134/v1 (PMC11703343; doi:10.21203/rs.3.rs-5632134/v1)
Supplement: Supplement 1 [file NIHPPRS5632134v1-supplement-1.pdf]

Supplementary Files

This is a list of supplementary files associated with this preprint. Click to download.

- Table1.docx
- Table2.docx
- Table3.tif
- Table4.docx
